# The Potential Role of Bacteriophages in the Treatment of Recalcitrant Chronic Rhinosinusitis

**DOI:** 10.3390/antibiotics10060675

**Published:** 2021-06-05

**Authors:** Saartje Uyttebroek, Jolien Onsea, Willem-Jan Metsemakers, Lieven Dupont, David Devolder, Jeroen Wagemans, Rob Lavigne, Isabel Spriet, Laura Van Gerven

**Affiliations:** 1 Department of Otorhinolaryngology, UZ Leuven, 3000 Leuven, Belgium; saartje.uyttebroek@uzleuven.be; 2 Department of Neurosciences, Experimental Otorhinolaryngology, Rhinology Research, KU Leuven, 3000 Leuven, Belgium; 3 Department of Trauma Surgery, UZ Leuven, 3000 Leuven, Belgium; jolien.onsea@uzleuven.be (J.O.); willem-jan.metsemakers@uzleuven.be (W.-J.M.); 4 Department of Development and Regeneration, Locomotor and Neurological Disorders, KU Leuven, 3000 Leuven, Belgium; 5 Department of Pneumology, UZ Leuven, 3000 Leuven, Belgium; lieven.dupont@uzleuven.be; 6 Department of Chronic Diseases and Metabolism, Respiratory Diseases and Thoracic Surgery, KU Leuven, 3000 Leuven, Belgium; 7 Pharmacy Department, UZ Leuven, 3000 Leuven, Belgium; david.devolder@uzleuven.be (D.D.); isabel.spriet@uzleuven.be (I.S.); 8 Department of Biosystems, Laboratory of Gene Technology, KU Leuven, 3000 Leuven, Belgium; jeroen.wagemans@kuleuven.be (J.W.); rob.lavigne@kuleuven.be (R.L.); 9 Department of Pharmaceutical and Pharmacological Sciences, Clinical Pharmacology and Pharmacotherapy, KU Leuven, 3000 Leuven, Belgium; 10 Department of Microbiology, Immunology and Transplantation, Allergy and Clinical Immunology Research Unit, KU Leuven, 3000 Leuven, Belgium

**Keywords:** chronic rhinosinusitis, recalcitrant, refractory, therapy-resistant, bacteriophages, phages, endolysins, phage therapy

## Abstract

Chronic rhinosinusitis is a common condition affecting 5–12% of the general population worldwide. In a limited number of cases, the disease is recalcitrant to medical and surgical interventions, causing a major impact on physical, social and emotional well-being and increasing pressure on healthcare systems. Biofilm formation and dysbiosis caused by *Staphylococcus aureus* and *Pseudomonas aeruginosa* play a role in the pathogenesis of recalcitrant chronic rhinosinusitis. In these cases, a promising treatment alternative is the application of bacteriophages, which are viruses that infect and lyse bacteria. In this review, we appraise the evidence for the use of bacteriophages in the treatment of recalcitrant chronic rhinosinusitis. Additionally, (dis)advantages of bacteriophages and considerations for implementation of phage therapy in otorhinolaryngology practice will be discussed.

## 1. Background

Chronic rhinosinusitis (CRS) is a common condition affecting 10.9% of the European population [1]. Sinonasal symptoms, according to the European Position Paper on Chronic Rhinosinusitis and Nasal Polyps 2020 (EPOS) [2], include nasal obstruction, nasal discharge (anterior or posterior nasal drip), facial pressure and a reduced sense of smell. In most cases, medical treatment with or without surgical intervention relieves these symptoms. However, in 15% of patients who underwent functional endoscopic sinus surgery (FESS) insufficient disease control can be obtained and symptoms persist, leading to therapy-resistant or recalcitrant rhinosinusitis (Table 1) [3].

Recalcitrant CRS is associated with a decreased quality of life (QoL), due to persistent sinonasal symptoms, recurrent need of systemic treatments (including corticosteroids) and sinus surgeries. Other comorbidities include sleep dysfunction, chronic pain, a negative impact on the lower airways and an increased risk of developing depression [4]. Besides the major impact of CRS on QoL, there is also an economic burden. The direct healthcare costs in Europe rise to approximately 2500 euro per patient per year and the indirect costs, due to missed workdays and decreased productivity, even exceed 5659 euro per patient per year [2].

### 1.1. Pathophysiology of Chronic Rhinosinusitis

**Figure 1:**
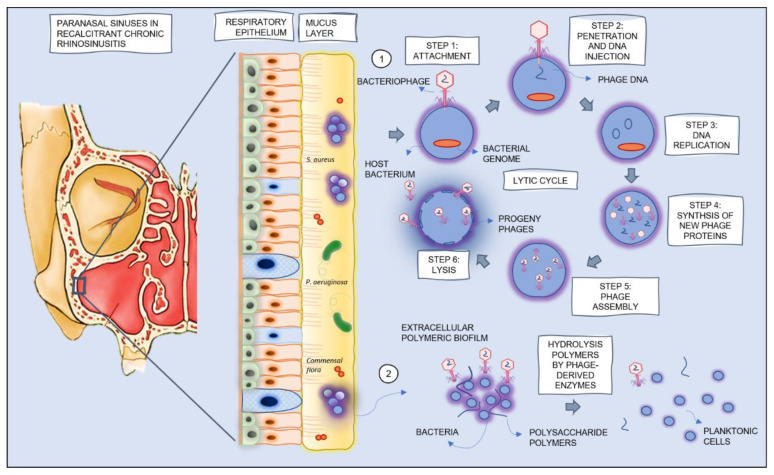
Schematic overview of part of the pathophysiology of recalcitrant CRS with biofilm formation, dysbiosis, overgrowth of *S. aureus* or *P. aeruginosa* and the theoretical mechanisms of action of bacteriophages in the treatment of recalcitrant CRS: (1) lysis of bacteria during the lytic phase of bacteriophage replication and (2) reduction of biofilm mass by hydrolysis of polymers by phage-derived enzymes.

CRS is a multifactorial chronic inflammatory disease with a complex pathophysiology, consisting of two major phenotypes: CRS with and without nasal polyps. Genetic predisposition, environmental factors (i.e., exposure to cigarette smoke) and microbial factors (i.e., changes in microbiome diversity, dysbiosis and biofilm formation) contribute to the onset and course of the disease, influencing epithelial barrier integrity and the adaptive immune response. The role of pathogenic bacteria is not clearly understood. Previous studies suggest a complex interaction between microbiota and the local immune system [2]. *S. aureus* colonizes the upper airways in one third of Europeans and is seen in 85% of patients with CRS and nasal polyps [5]. Moreover, in 9% of CRS patients and in 49% of CRS patients with cystic fibrosis (CF), *P. aeruginosa* can be isolated from the nasal cavity [6]. Colonization of the upper airway by *S. aureus* or *P. aeruginosa* is associated with recurrent infections, a poor outcome after FESS and a poor overall prognosis, often leading to therapy failure and recalcitrant disease (Figure 1) [6,7,8].

*S. aureus* and *P. aeruginosa* have the ability to form a biofilm, a complex extracellular polymeric matrix consisting of diverse polysaccharides, proteins, lipids and extracellular DNA. Biofilm formation makes bacteria 100–1000 fold more resistant to antibiotics by reducing the ability of antibiotics to penetrate the bacteria, by expressing resistance genes and by inducing changes in cellular metabolisms [9,10]. The last decade, different antibiofilm agents have been proposed to treat difficult-to-treat polymicrobial biofilms, e.g., colloidal silver, probiotic irrigations, Manuka honey, surfactants (i.e., baby shampoo) and xylitol [11]. However, data on these biofilm treatments remain limited and the additional benefits remain unclear. The use of these antibiofilm agents, except for xylitol, in the treatment of CRS is not supported by the EPOS guidelines [2].

For these bacterial species, the increasing rate of antibiotic resistance is alarming in all parts of the world. According to the World Health Organization, antibiotic resistance is one of the biggest threats to global health [12]. Consequently, growing numbers of infections will be difficult to treat, leading to longer hospital stays, higher medical costs and increased morbidity and mortality. Unfortunately, the development of new antibiotics is not as fast as the clinical needs, increasing the danger of entering a post-antibiotic era. For these reasons, scientists are urgently exploring new antimicrobial strategies.

### 1.2. Role of Bacteriophages or Phage-Derived Endolysins in the Treatment of Recalcitrant CRS

Bacteriophages (or phages) are naturally occurring viruses that specifically target, multiply within and lyse bacteria. Phages have (re)gained interest over the past decade. Historically, the discovery and first application of bacteriophages dates back to the beginning of the 20th century. After the discovery of penicillin by Fleming, research and development of bacteriophage therapy was largely abandoned. However, this was not the case in some centers in the former Soviet Union and Eastern Europe, where phage therapy remained common practice. Today, mainly due to the increasing threat of antimicrobial resistance, the concept of phage therapy is regaining popularity in Western Europe [11].

Bacteriophages can generally be divided in two groups, based on their mechanism of infection: lytic and temperate phages. Strictly lytic phages bind to their specific host bacteria, inject their genetic material and replicate within the bacterium. Afterwards, the host cell is lysed and progeny phages are released in the environment, where they can infect adjacent bacteria (Figure 1) [6]. Temperate phages integrate their genetic material in the bacterial genome as prophages or remain present as plasmid-like molecules. Under favorable conditions, prophages can be activated and continue the lytic cycle. This cycle is also known as the lysogenic cycle. Temperate phages are known to lead to horizontal gene transfer or ‘transduction’ of resistance mechanisms or virulence factors, therefore only strictly lytic phages should be used as therapeutic agents [13]. The potential to self-amplify within the host bacterial cell is one of the main advantages contributing to the therapeutic potential of phages. Another important characteristic is their specificity for host cells, implying that phages do not harm normal bacterial flora or eukaryotic (human) cells. Moreover, bacteriophages can also contain enzymes on their tail proteins, such as polysaccharases, polysaccharide lyases and depolymerases, which have the ability to degrade and to penetrate the biofilm matrix, making bacteria more vulnerable to phage infection and to classic antibiotics (Figure 1) [14].

Lastly, phage-derived endolysins (or shortly: lysins) are emerging as an alternative therapeutic agent in the treatment of therapy-resistant infections. Endolysins are phage-encoded enzymes, which are produced by the end of the lytic cycle. These lysins have the ability to hydrolyze and disrupt the host bacteria’s peptidoglycan layer, leading to lysis of the bacterium. Interestingly, isolated lysins have several advantages when compared to intact bacteriophages, i.e., bacterial lysis is less dependent on multiplicity of infection, no resistance has been reported to date, lysins are relatively stable when compared to phages and they have well defined pharmacokinetics [15].

Bacteriophages can be considered in recalcitrant chronic rhinosinusitis as an antibiofilm and antimicrobial agent, with or without concomitant use of antibiotics. The aim of this narrative review is to summarize the current evidence regarding the use of bacteriophages in the treatment of recalcitrant chronic rhinosinusitis, based on preclinical (in vitro and animal studies) and clinical trials.

## 2. Preclinical Studies

### 2.1. In Vitro Studies Using Ex Vivo Bacterial Strains from CRS Patients

#### 2.1.1. Activity of Bacteriophages and Phage-Derived Enzymes against *S. aureus*

In 2014, the in vitro activity of bacteriophages against *S. aureus* strains from CRS patients was examined for the first time [16]. Sixty-six *S. aureus* isolates, obtained from sinonasal swabs of CRS patients, were tested for phage sensitivity to a single phage (SA1) and a phage cocktail (CT-SA), consisting of four *S. aureus* specific phages. Ninety percent of the strains were lysed by SA1 and 94% were lysed by CT-SA. The biofilm mass was measured using a minimum biofilm eradication concentration (MBEC) assay and fluorescent scanning microscopy. The biofilm mass was significantly reduced in 80% of the tested strains after application of CT-SA. The emergence of bacteriophage-insensitive mutants was higher in the SA1-treated group, suggesting that application of a single phage is inferior to application of a phage cocktail.

More recently, Drilling et al. tested 61 clinical CRS *S. aureus* isolates for phage sensitivity to two single phages P68, K710 and the cocktail of these phages, NOV012 [8]. Sensitivity towards the single phages P68 and K710 was 74% and 59%, respectively. Eighty-five percent of the isolates were sensitive to the NOV012 cocktail among which all tested methicillin-resistant (MRSA) strains. Likewise, Zhang et al. collected 65 clinical *S. aureus* isolates from CRS patients [7]. The incidence of antibiotic resistance of the clinical strains was as high as 90.7%, with 20% showing resistance to three or more antibiotics. The antibiotic resistant *S. aureus* strains were sensitive to single phages Sa87 and Sa83 in 71.1% and 69.4% respectively. There was no significant difference in phage sensitivity between the antibiotic sensitive and antibiotic resistant strains, showing that phage sensitivity is not related to antibiotic sensitivity. The Sa83 and Sa87 bacteriophages were able to reduce all *S. aureus* strains. In another study, the phage cocktail AB-SA01 was tested against 15 *S. aureus* CRS isolates, resulting in a sensitivity of 80% [10].

In 2018, Bachert C. et al. obtained polyp tissue from the ethmoidal sinuses of 17 CRS patients during sinus surgery [5]. *S. aureus* was isolated in nine cases and Interleukin-5 (IL-5) levels were more than threefold higher in *S. aureus* carriers. IL-5 is an inflammatory marker that plays a role in the differentiation, activation and survival of eosinophils and is important in the pathophysiology of chronic rhinosinusitis with nasal polyps. The combination of antibiotics and ISP (intravenous staphylococcal phage), a *S. aureus* specific phage, could reduce IL-5 levels after 24 and 72 h.

Drilling et al. tested the ability of P128, a bacteriophage-derived muralytic enzyme, to degrade biofilms produced by *S. aureus,* isolated from clinical strains of CRS patients [17]. Various concentrations of P128 were tested and biofilm eradication was measured using MBEC and Alamar Blue (AB) assay and visualized with scanning laser microscopy. Interestingly, P128 was able to significantly reduce biofilm mass in up to 95.5% of all tested clinical strains.

#### 2.1.2. Activity of Bacteriophages against *P. aeruginosa*

Forty-seven *P. aeruginosa* isolates from CRS patients, with and without CF, were tested for phage sensitivity to four single phages (Pa193, Pa204, Pa222 and Pa223) and to these phages combined in a phage cocktail (CT-PA) [6]. Eighty-nine percent of the isolates were lysed by the CT-PA cocktail, whereas 53–73% of the isolates were lysed by the individual phages. The authors also found a significant reduction of biofilm mass, assessed with the microtiter dish biofilm assay, after 24 and 48 h of treatment with CT-PA, Pa222 and Pa223. Remarkably, the antibiofilm activity of CT-PA was not affected by multidrug resistance or presence of CF.

More recently, a study was conducted to assess the microbiology of the upper airways in CRS patients and the sensitivity of the bacteria to phages [18]. Fifty CRS patients, who had undergone endoscopic sinus surgery, were included leading to the identification of 97 bacterial isolates. The most commonly isolated pathogens were *S. aureus*, *Staphylococcus epidermidis*, *Haemophilus influenzae* and *P. aeruginosa*. Forty-six percent of the patients carried antibiotic-resistant bacteria and cultures of 28% of the patients were not sensitive to amoxicillin-clavulanic acid. Eighty percent of the isolates, on the other hand, were sensitive to the bacteriophages of the collection used in this study.

An overview of all in vitro studies is summarized in Table 2.

### 2.2. Animal Studies

In 2014, Drilling et al. developed a sheep model of rhinosinusitis using mini-trephinations to gain access to the frontal sinuses [9]. Twenty-seven sheep were included and the sinuses were inoculated with *S. aureus*. Afterwards, the frontal sinuses were flushed with either a *S. aureus* specific phage cocktail (CT-SA) or EDTA (ethylenediaminetetraacetic acid), which acts as a metal chelator with antibiofilm characteristics and the combination of CT-SA and EDTA. Safety was assessed after five days of treatment, by histological examination of the respiratory epithelium using scanning electron microscopy. Efficacy was determined by imaging of the biofilm mass using BacLight staining. Histological examination showed no damage to the sinonasal mucosa after phage therapy and biofilm mass was significantly reduced after CT-SA treatment, demonstrating that a short course of phage therapy is safe and effective. No synergy could be observed between CT-SA and EDTA.

The same sheep model was subsequently used to determine the safety of long-term phage applications [8]. The frontal sinuses were inoculated with *S. aureus* and flushed with a phage cocktail, NOV012, for 20 days. The general well-being, including appetite, of the sheep did not change during phage therapy. During the experiment, no active phages were detected in serum. Afterwards, histological examination showed no differences in inflammation, edema, fibrosis and the presence/absence of goblet cell hyperplasia between the intervention group and the control group, also indicating the safety of long-term phage therapy.

Recently, the sheep model was adapted to determine the mechanisms of action of a *P. aeruginosa* specific phage cocktail, CT-PA [19]. A *P. aeruginosa* strain was obtained from an endoscopically guided sinus swab from a CRS patient and was instilled in the frontal sinuses of thirty-two sheep. The sheep were divided in four treatment groups (saline, 4 × 10^8^ plaque forming units (PFU)/mL CT-PA, 4 × 10^9^ PFU/mL CT-PA and 4 × 10^10^ PFU/mL CT-PA; for seven days). Blood sampling during phage therapy showed no significant changes in hematology and biochemistry. After phage therapy, the biofilm mass was assessed, showing a significant reduction in mean biofilm mass in the CT-PA treated groups when compared to saline rinsing. There was no significant difference between the various concentrations. Histopathology showed no significant differences. The degree of epithelial hyperplasia was significantly lower in the CT-PA treated group, compared to placebo.

The intranasal application of a bacteriophage-derived enzyme was tested for the first time in an animal model a decade ago. Fenton et al. determined whether a single application of a bacteriophage-derived lysin (CHAPk) could reduce the bacterial count of a *S. aureus* strain [20]. Therefore, they instilled *S. aureus* isolates in the nares of 14 mice. Afterwards, CHAPk was applied in seven mice, the other mice were treated with a buffer solution and served as a control group. One hour post-treatment, the authors saw that the lysin was highly active against *S. aureus* and was able to reduce the bacterial cell count by two log units.

An overview of these animal trials is provided in Table 3.

## 3. Clinical Trials

### 3.1. Safety of Phage Therapy in Recalcitrant CRS

In 2018, a phase I safety trial was conducted, involving 21 healthy (nasal) *S. aureus* carriers [21]. All subjects received three therapeutic regimens: a staphylococcal monophage, Eliava’s pyophage cocktail (effective against *Staphylococcus* spp., *Streptococcus* spp., *E. coli*, *P. aeruginosa* and *Proteus* spp.) and placebo. Phage therapy or placebo suspension (10 mL) was applied orally in ten participants and intranasally in eleven participants, three times a day for two consecutive days. Intranasal and oral application of bacteriophages were not associated with significant changes in blood values (including biochemistry, hematology, renal and liver function) when compared to placebo. Four subjects, of which one in the placebo group, mentioned adverse events during oral treatment. Relevant adverse events were gastrointestinal problems (vomiting, loose stool, gastric acidity and mild epigastric pain), back pain and low grade fever. None of the adverse events were related to the treatment, according to the clinicians. No adverse events were observed during intranasal treatment.

In 2019, another small phase I trial was conducted to investigate intranasal application of a *S. aureus* specific phage cocktail, AB-SA01, in patients with recalcitrant CRS [10]. Nine CRS patients, in whom medical and surgical treatment had failed and who had positive *S. aureus* cultures sensitive to AB-SA01, were included and were divided in three cohorts. The participants received high-volume intranasal irrigations with AB-SA01 at different concentrations (3 × 10^8^ PFU/mL for 7 days, 3 × 10^8^ PFU/mL for 14 days and 3 × 10^9^ PFU/mL for 14 days). No changes in vital signs and biochemistry were noted. Mild adverse events were seen in six participants, including diarrhea, epistaxis, oropharyngeal pain, cough and rhinalgia. These complaints resolved by the end of the trial. Intranasal irrigations were considered to be safe and well-tolerated with no serious adverse events.

### 3.2. Efficacy of Phage Therapy in Recalcitrant CRS

The first human experimental trial, with observational design, investigating the efficacy of phage therapy in CRS patients was already published in 1956 [22]. Sixty CRS patients, colonized with *S. aureus*, received a bacteriophage lysate (A-1 or B-7) by nasal inhalation using a nebulizer. The aerosol consisting of A-1 lysate was administered intranasally until objective and subjective relief of all symptoms was achieved. When there was insufficient clinical improvement, B-7 lysate was added. Afterwards, a monthly maintenance or booster dose was administered. The mean duration of the treatment was two to six weeks. The clinical results were graded as excellent, good, fair and poor in respectively 45%, 33%, 17% and 5% of the cases.

The Institute of Immunology and Experimental Therapy in Poland published an overview of their experience with phage therapy in the treatment of therapy-resistant infections. The authors included 1307 patients with suppurative bacterial infections in which antibiotic therapy had failed (in the years 1987–1999) [23]. Forty-six of these patients suffered from rhinosinusitis and were treated with phage therapy. The therapy depended on the causative pathogen and its sensitivity to the available phages from the phage collection. Bacteriophages were orally or intranasally administered. Full recovery, with complete eradication of the causative bacteria, was observed in 83% of the cases. Improvement of the symptoms, but without full eradication of the bacteria, was observed in 7% of patients. At the Phage Therapy Unit in Wroclaw, a retrospective trial was conducted to investigate the induction of antiphage antibodies during phage therapy [24]. Twenty-five CRS patients underwent phage therapy, consisting of local (sinus irrigations or nasal spray) or a combined oral and local treatment. The selected phage depended on the phage sensitivity of the associated causative pathogen. Before, during and after phage therapy, sera were obtained from these patients. Before phage therapy phage inactivation rates, defined as inactivation of specific phages by the patient’s serum (K), were low and they increased significantly during phage therapy. Twenty-four percent of the patients had a high phage inactivation rate. Thirty-three percent of this population had a positive response, whereas 27.8% of patients with a low K rate had a positive response, suggesting that the level of phage neutralization does not affect the outcome of phage therapy. A few years later, a randomized controlled trial was conducted in Russia, comparing the efficacy of phage therapy to antibiotics in the treatment of acute rhinosinusitis [25]. Fifty patients with acute maxillary sinusitis were admitted to the day care hospital and underwent puncture and rinsing of the maxillary sinus. The intervention group, consisting of 37 patients, received maxillary rinsing with the Eliava Pyophage cocktail and afterwards they continued oral phage therapy for ten days. Twenty patients in the control group received rinsing of the maxillary sinus with a saline solution and afterwards they received a second generation cephalosporin for ten days. After the treatment, the efficacy of the pyophage was comparable to that of second generation cephalosporins. The effect of the phage therapy was seen after 3–4 days and of the antibiotic course after 4–5 days.

As mentioned previously, Ooi et al., conducted a phase I clinical trial in nine CRS patients [10]. After phage therapy, all patients had reduction in *S. aureus* growth with negative cultures in two out of nine patients. The endoscopic Lund-Kennedy score improved in all patient cohorts, with greatest improvement in patients receiving 3 × 10^9^ PFU/mL AB-SA01 for 14 days.

An overview of these clinical trials is summarized in Table 4.

## 4. Interpretation of the Available (Pre)Clinical Data

Overall, 53–90% of the ex vivo isolates (mostly *S. aureus* and *P. aeruginosa*) from CRS patients were sensitive in vitro to the tested single phages and 85–94% of the isolates were sensitive to the phage cocktails. Even (multi)drug-resistant strains from CRS patients, including MRSA, were lysed by bacteriophages, showing that their mechanism of action is not influenced by antibiotic resistance [6,7,8,18]. Interestingly, bacteria isolated from the upper airway of patients with cystic fibrosis were also sensitive to the bacteriophages [6]. Moreover, several trials have demonstrated that phages are able to reduce biofilm mass, produced by *S. aureus* and *P. aeruginosa*, in CRS isolates [6,8,16,17,19].

Animal and human trials were conducted to assess the safety of phage therapy in CRS patients. A sheep model demonstrated that local application of bacteriophages to the frontal sinuses is safe and does not damage the respiratory epithelium after 14 days of treatment [8,9,19]. Observational human clinical trials have demonstrated that, both oral and intranasal, phage therapy is generally well-tolerated and is not associated with serious adverse events, changes in blood values or changes in vital signs [10,21,22,23,24,25].

Unfortunately, high-quality efficacy trials in patients with chronic rhinosinusitis remain lacking and the results are variable. A recently published phase I trial showed reduction of symptoms and improvement of endoscopic appearances in all CRS patients after seven days of nasal rinsing. The causative pathogen was eradicated in two out of nine patients. The sample size was too small to carry out statistical analyses [10]. Patient registries, set up in Eastern Europe, showed varying results [23,24]. Neither of the studies had a placebo or control group. Noteworthy is that promising in vitro results do not necessarily predict a good clinical outcome, as chronic rhinosinusitis has a complex etiology and the role of bacteria is not fully understood. It is unclear whether improvement of the microbiome might alleviate all CRS associated symptoms. Larger and qualitative human trials should be carried out to determine the efficacy of phage therapy in the treatment of recalcitrant CRS.

## 5. (Dis)Advantages of Phage Therapy versus Antibiotics

**Figure 2:**
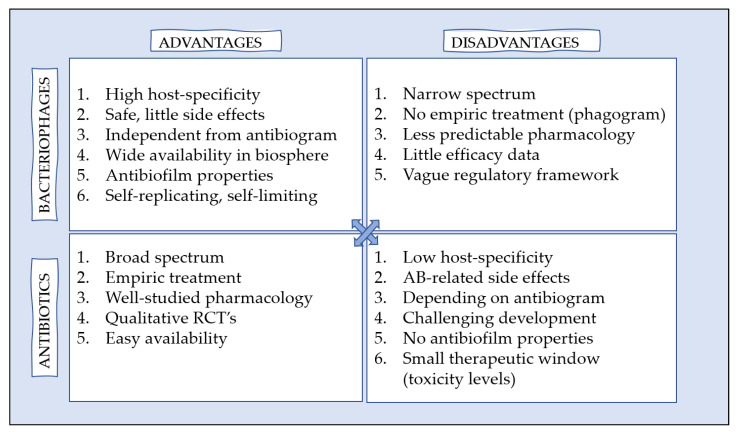
Advantages and disadvantages of bacteriophages and antibiotics.

Bacteriophages have both distinct advantages and disadvantages when compared to antibiotics (Figure 2). First, bacteriophages bind specifically to their host bacteria and thereby do not harm the surrounding (human) tissues and commensal flora [26,27]. This high specificity results in limited, undesired side effects [28]. In contrast, antibiotics also target healthy tissues and are associated with antibiotic-related side effects (e.g., diarrhea, hearing loss, nephrotoxicity secondary infections, etc.), sometimes requiring therapeutic drug monitoring [28]. However, the narrow host range of phages impedes empiric treatment, necessitating phage sensitivity testing before the start of the treatment (causing a delay between diagnosis and start of the treatment). Combining single phages in phage cocktails can increase the host range and may be beneficial towards limiting phage resistance emergence [29].

The major concern regarding antibiotics is the rapid rise of multidrug resistant bacteria. Developing new antibiotics is a time-consuming and expensive process. As bacteriophages use different mechanisms of action, they are able to lyse antibiotic-resistant bacteria. New bacteriophages can also be relatively easily isolated from the environment (ground water, soil, etc.) [29]. The development of bacterial resistance against bacteriophages can occur, but has not been studied in detail in human trials. Simultaneous use of bacteriophages and antibiotics might decrease the risk of induction of antibiotic resistance [30]. Moreover, the formation of neutralizing antiphage antibodies is not fully understood. The presence of antiphage antibodies in patients’ serum, before or after phage therapy, has been considered to be a limitation to the success of phage therapy, especially after repeated administration. Lusiak et al. could not find a correlation between the presence of antiphage antibodies and the clinical outcome of phage therapy [31,32]. This topic should be further evaluated.

Second, general rules of pharmacology do not apply to bacteriophages as their replication depends on the bacterial density [26]. Bacteriophages are self-replicating in the presence of their host bacteria and, after the infection has resolved they are self-limiting [27]. Besides, the titer of phages (e.g., in a stock solution) is variable and can be influenced by external factors, for instance temperature and storage materials [29]. These characteristics make it hard to predict individual patient outcomes.

As mentioned earlier, bacteriophages can produce biofilm-degrading enzymes and are thereby able to reduce biofilm mass, whereas biofilm formation limits the penetration and efficacy of antibiotics [14].

Antibiotics are well-studied and widely available, making them easily accessible for the general population. Implementation of phage therapy requires multidisciplinary cooperation and setting up a regulatory framework. Based on their characteristics, simultaneous use of antibiotics and phage therapy can possibly have additive or synergistic effects [27].

## 6. Considerations before Implementation of Phage Therapy in Recalcitrant Chronic Rhinosinusitis

### 6.1. Vision on Phage Therapy in Clinical Practice

Several considerations should be taken into account before applying phage therapy in clinical practice or before setting up clinical trials.

First, a multidisciplinary team of experts (e.g., ENT specialists, infectious disease specialists and microbiologists), should agree on the indication for phage therapy. Phage therapy should be reserved for “last resort” patients with difficult-to-treat chronic rhinosinusitis in which all previous treatments have failed. Second, the causative pathogen should be isolated before the start of the treatment, using an endoscopically guided swab from the middle meatus. Phage sensitivity should be tested in vitro. The therapeutic phage should be purely lytic and the use of temperate phages should be avoided, to reduce the risk of horizontal gene transfer [3]. Hence the concerns regarding temperate phages, next generation sequencing techniques make it possible to modify temperate phages to become purely lytic. As temperate phages are more abundant in nature and more easily to isolate, phage engineering is an interesting entity. Although using temperate phages sounds promising, the clinical outcomes, the immunogenicity and effects on gene transfer remain unknown [13].

Third, based on the current data, local application might be preferred over systematic administration to circumvent potential systemic side effects of phage therapy (i.e., induction of antiphage antibodies). Access of local products to the paranasal sinuses depends on the anatomical appearances of the sinuses and the delivery method [3]. Previous sinus surgery enhances the delivery of topical drugs into the sinuses. The optimal delivery method (e.g., high volume rinsing, spray, nasal inhalation and endoscopy-guided rinsing) and duration of treatment have not yet been established in a systematic manner. Furthermore, phage stability and the active phage titer in the transport medium should be assessed. Currently, the optimal concentration of the phage solution still has to be elucidated, but is approximately between 10^7^ and 10^9^ PFU/mL [3].

### 6.2. Regulatory Framework

As phage therapy is potentially beneficial in the treatment of recalcitrant CRS, scientists should be encouraged to set up larger, qualitative human clinical trials. Unfortunately, regulatory frameworks are lacking, discouraging scientists and funding instances to implement phage therapy. In Western Europe, phage therapy has been performed as “compassionate use” treatment under the legislation of the Declaration of Helsinki (art. 37 June 1964), in patients with therapy-resistant bacterial infections in which all treatments have failed, after informed consent of the patient [33].

In Belgium, applying phage therapy for difficult-to-treat bacterial infections within the framework of magistral preparations was approved in 2019 [33]. In this regard, the University hospitals of Leuven (UZ Leuven) set up a multidisciplinary phage task force consisting of ENT specialists, infectious diseases specialists, microbiologists, scientists and pharmacists. The task force evaluates and selects patients who might benefit from phage therapy. The causative pathogen will be tested against an available phage panel. If the strain is sensitive to an available phage from the collection, phage therapy can start. The task force ensures that the application protocol (route of administration, dose, frequency of administration/application and duration) and follow-up schedules are standardized.

A better understanding of phage therapy will be a key component in the battle against multidrug resistant bacteria.

## 7. Conclusions

Bacteriophages are (re)gaining interest in the treatment of difficult-to-treat infections. In vitro studies have demonstrated that bacterial strains from patients with chronic rhinosinusitis are sensitive to bacteriophages in most cases, independent from their sensitivity to antibiotics and that bacteriophages are able to reduce biofilm mass produced by *S. aureus* and *P. aeruginosa* in these patients. Systemic and intranasal application of bacteriophages is generally considered safe without occurrence of major side effects. In the future, high-quality human trials—in accordance with good clinical practice guidelines—are needed to demonstrate the efficacy of phage therapy in recalcitrant CRS and to investigate the possibility of synergistic effects between bacteriophages and antibiotics.

## Figures and Tables

**Table 1:** Definition of chronic rhinosinusitis and recalcitrant chronic rhinosinusitis, according to EPOS 2020 guidelines.

Chronic rhinosinusitis [2]	Presence of two or more symptoms, one of which should be either nasal blockage/obstruction/congestion or anterior/posterior nasal drip: ±facial pain/pressure, ±reduction or loss of smell; for >12 weeks
Recalcitrant chronic rhinosinusitis [2]	Patients who have persisting symptoms of rhinosinusitis despite appropriate treatment (recommended medication and surgery)

**Table 2:** Overview of in vitro studies demonstrating the sensitivity and efficacy of phages or phage-derived enzymes to ex vivo isolates from CRS patients.

Authors (Year)	Isolates Sinonasal Swabs	Included CRS Patients	Phage	Phage Sensitivity	Efficacy
Drilling A. et al. (2014) [16]	*S. aureus*	66	CT-SA, cocktail SA1, single phage	94% 90%	Biofilm mass reduction: 80% after CT-SA application
Drilling A. et al. (2016) [17]	*S. aureus*	NS	P128, bacteriophage derived muralytic enzyme	NS	Biofilm mass reduction: 95.5%
Fong S. et al. (2017) [6]	*P. aeruginosa*	47 ^1^	Pa193, single phage Pa204, single phage Pa222, single phage Pa223, single phage CT-PA, cocktail	73% 53% 73% 71% 85%	Significant biofilm mass reduction after CT-PA, Pa222 and Pa223
Drilling A. et al. (2017) [8]	*S. aureus*	61	P68, single phage K710, single phage NOV012, cocktail	74% 59% 85%	NS
Bachert C. et al. (2018) [5]	*S. aureus*	9 ^2^	ISP, single phage	NS	Reduced IL-5 levels after 24 and 72 h, no significant changes compared to antibiotics
Zhang G. et al. (2018) [7]	*S. aureus*	65	Sa83, single phage Sa87, single phage	69% 71%	NS
Szaleniec J. et al. (2019) [18]	Different pathogen ^3^	50	Sensitive phage from collection Biophage Pharma, NS	80%	NS
Ooi M. et al. (2019) [10]	*S. aureus*	15	AB-SA01, cocktail	80%	NS

Abbreviations: NS = not specified. ^1^ CRS patients with or without cystic fibrosis. ^2^ Samples collected during functional endoscopic sinus surgery. ^3^ Different pathogen including *S. aureus*, *S. epidermidis*, *P. aeruginosa* and *H. influenzae*.

**Table 3:** Overview of animal studies investigating the safety and efficacy of bacteriophages or phage-derived enzymes in the treatment of chronic rhinosinusitis.

Authors (Year)	Pathogen	Animal Model + Application Method	Included Subjects	Phage	Safety	Efficacy
Fenton M. et al. (2010) [20]	*S. aureus*	Mice, intranasal instillation	14	Phage lysin CHAP_k_ from bacteriophage K	NS	Two-log reduction in *S. aureus* cells 1 h after single application
Drilling A. et al. (2014) [9]	*S. aureus*	Sheep, frontal rinsing via mini-trephinations	27	CT-SA cocktail	No histological changes to frontal sinus mucosa	Significant reduction of biofilm mass
Drilling A. et al. (2017) [8]	*S. aureus*	Sheep, frontal rinsing via mini-trephinations	21	NOV012 cocktail	No histological changes to frontal sinus mucosa	NS
Fong S. et al. (2019) [19]	*P. aeruginosa*	Sheep, frontal rinsing via mini-trephinations	32	CT-PA cocktail	No histological changes to frontal sinus mucosa	Significant reduction of biofilm mass

Abbreviations: NS = not specified.

**Table 4:** Overview of clinical trials investigating the safety and efficacy of phages or phage-derived enzymes in the treatment of (recalcitrant) rhinosinusitis.

Authors (Year)	Study Type	Participants	Pathogen	Therapeutic Regimen	Phage	Safety	Efficacy
Mills E. et al. (1956) [22]	OBS	Recalcitrant CRS (*n* = 60)	*S. aureus*	Nasal nebulizer	Phage lysate A-1 and B-7	No reported AE	Clinical improvement: Excellent: 45%, Good: 33%, Fair: 17%, Poor: 5%
Weber-Dabrowska et al. (2000) [23]	OBS	Supparative sinusitis (*n* = 46)	Different pathogen ^1^	Oral or nasal drops, NS	Sensitive phage from collection, NS	NS	Clinical improvement: Full recovery: 83%, Marked: 7%, No effect: 11%
McCallin S. et al. (2018) [21]	OBS	Healthy carriers (*n* = 21)	*S. aureus*	Oral (*n* = 10) or nasal application (*n* = 11)	Staphylococcal monophage (*n* = 21), Pyophage cocktail (*n* = 21), Placebo (*n* = 21)	No reported AE after nasal therapy, mild AE ^2^ in 4 subjects after oral therapy. No changes in blood values.	NS
Kryukov A. et al. (2019) [25]	RCT	Acute maxillary sinusitis (*n* = 58)	Different pathogen ^3^	Peroperative nasal rinsing, followed by oral treatment (*n* = 38); Second generation cephalosporin (*n* = 20)	Polyvalent Pyophage	No reported AE	Clinical improvement: No significant changes between both groups after 10 days of treatment
Ooi M. et al. (2019) [10]	OBS	Recalcitrant CRS (*n* = 9)	*S. aureus*	Intranasal high-volume irrigations	AB-SA01, cocktail	Mild AE ^4^ in 3 patients, all of them resolved by the end of the trial. No changes in vital signs or blood values.	Improved LKS in all patients. Reduction bacterial load: 100%. Eradication bacteria: 22%
Lusiak M. et al. (2020) [24]	OBS	CRS (*n* = 25)	Different pathogen ^5^	Nasal (*n* = 4) or nasal + oral application (*n* = 21)	Sensitive phage from collection	NS	Clinical response: Positive: 32%, inadequate: 68%

Abbreviations: OBS = observational study; RCT = randomized controlled trial; CRS = chronic rhinosinusitis; AE = adverse events; *n* = number; NS: not specified; LKS = Lund-Kennedy Score. ^1^ Different pathogen including *S. aureus*, *E. coli, Klebsiella*, *Proteus* and *P. aeruginosa*. ^2^ Mild adverse events including vomiting, loose stool, gastric acidity, mild epigastric pain and low grade fever. ^3^ Different pathogens including *S. pneumoniae*, *S. aureus*, *H. influenzae* and hemolytic streptococci. ^4^ Mild adverse events including diarrhea, epistaxis, oropharyngeal pain, cough and rhinalgia. ^5^ Different pathogen including *S. aureus*, *P. aeruginosa*, *Klebsiella pneumoniae* and *E. coli.*
